# The impact of positive activities on mental health: the mediating role of positive emotion

**DOI:** 10.3389/fpubh.2024.1474544

**Published:** 2024-12-10

**Authors:** Yanting Wang, Yuanyang Wu, Qianqian Xu, Si Fan, Jinwen Hu, Dongdong Zou, Xinping Zhang

**Affiliations:** School of Medicine and Health Management, Tongji Medical College of Huazhong University of Science and Technology, Wuhan, Hubei, China

**Keywords:** positive activities, mental health, positive emotion, positive-activity model, mediating role

## Abstract

**Objective:**

Mental health has become a widely concerned topic worldwide. However, the impact and mechanism of positive activities on mental health still needed to be explored. This study aimed to apply the positive-activity model to investigate the effect of participation in positive activities on mental health and the mediating role of positive emotion.

**Methods:**

This study used data from the 2021 China Comprehensive Social Survey (CGSS) and included 2,581 individuals. Ordinary Least Squares (OLS) and a three-step method was used for analysis.

**Results:**

The average of positive activities was 15.83. The positive activities affected positively mental health (β = 0.0132, *p* < 0.001). The positive emotion played a mediating role (β =0.2281, *p* < 0.001). The effect of positive activities on mental health was significant in older adults group (β = 0.024, *p* < 0.001), female (β = 0.015, *p* < 0.01) and male group (β = 0.01, *p* < 0.01), unmarried/divorced/widowed group (β = 0.024, *p* < 0.01), cohabitation/first marriage with spouse/remarriage with spouse/separation without divorce group (β = 0.010, *p* < 0.001), middle(β = 0.013, *p* < 0.05), and upper-middle-level SES group (β = 0.054, *p* < 0.001).

**Conclusions:**

We concluded that the participation level of positive activities still needs to be improved and positive activities improve mental health through positive emotion, which implied that positive activities, as an easily implementable measure, should be greatly encouraged in mental health policies. And older adults, female, people without spouse, middle and upper-middle-income individuals need to be paid more attention.

## 1 Introduction

As perceptions of health have changed, mental health has become as important as physical health and cannot be ignored (1), which affects personal development, quality of life, daily life functions, etc. (2, 3). However, poor mental health has become a global public health issue. According to the report released by the World Health Organization (WHO) in 2022, 970 million people globally suffered from mental disorders in 2019 (4). Mental illness can lead to serious economic, social, and political problems (5). Addressing mental health issues has become a key priority for public health programs (6). Therefore, it is necessary to pay attention to the topic of mental health.

The positive activity model proposed by Lyubomirsky and Layous explains the relationship between positive activity and wellbeing, and suggests that positive emotion can mediate the relationship between positive activity and wellbeing (7). Positive activity is defined as simple, low-cost, self-managed, purposeful, and regular practices, which can enhance the sense of wellbeing (7). Positive activities include various forms such as communication with friends, exercise, and dancing (8). The positive activity model has been used to explain the impact of different forms of positive activity on individuals' thoughts. For example, nurses using their strengths at work can reduce their depression levels (9). People who participate in square dancing for a longer period of time have a better overall feeling of life quality (10). These studies suggest that certain positive activities can increase healthy thoughts. Therefore, we argued that positive activities can affect mental health via positive emotion based on the positive-activity model.

Participating activities is widely advocated and practiced globally to promote health (11, 12). However, the effect of participating in positive activities on mental health still needs to be explored. Previous researches shown that positive activities have an impact on mental health. But these researches mainly focused on a single type of positive activities, with physical activities and social activities being the most studied (13–16). For example, Hou et al. showed that a higher level of physical activity was associated with better mental health (15); some studies suggested that people who engage in more social activities have better mental health (16). There are also a few studies on the impact of various types of leisure activities on mental health, in which various types of activities included physical exercise, social activities, mental activities, reading, sewing, etc. (16–19). However, despite some of these researches, in terms of the type of positive activities, activities such as reading, watching TV, and shopping have received little attention. Thus, it is necessary to include these forms of activities in the study. In addition, existing researches on the impact of activities such as reading and watching TV on mental health has yielded inconsistent results (18, 20, 21), which indicated that more evidence is still needed. Therefore, it is urgent and valuable to assess the impact of overall level of participation in positive activities and various types of positive activities on mental health for enriching relevant research evidence, enriching the evidence-based application of positive psychology and improving mental health policies.

Although the correlation between participation in positive activities and mental health has been studied, the mechanism still needs to be explored. Previous studies have shown that positive emotion can mediate positive activities and positive outcomes. For example, Pradhan et al. used the positive activity model to explore the positive impact of spirituality at workplace on engagement and the mediating role of emotional intelligence (22). Zhang et al. also used this model to explore the impact of square dancing on the quality of life of older adults and the mediating role of their own aging attitudes (10). However, few scholars have studied whether positive emotion can play a mediating role between positive activities and mental health based on the positive-activity model. Previous studies have suggested that positive emotion may play a mediating role. On the one hand, previous studies have indicated the impact of positive activities on positive emotion. For example, Chen et al. believed that participating in leisure activities promoted the accumulation of psychological resources such as increasing optimism and their research showed that participating in leisure activities was positively correlated with a higher level of emotional health (23). Ba et al. found an association between time allocated to daily activities/social interactions and the duration of positive emotion (24). A qualitative study in Canada suggested that participating in various activities can help individuals focus on their own time, generate a series of positive emotion (25). On the other hand, positive emotion can improve mental health. Positive emotion is a short-term feeling but the benefits brought by it can accumulate and compound, bringing changes in people's thoughts, behaviors and psychological responses, predicting thriving mental health (26, 27). Therefore, our study explored the potential mechanisms of the effect of positive activities on mental health, with a view to providing a theoretical basis for alleviating mental health problems.

In summary, this study aimed to apply the positive-activity model to investigate the effect of participation in multiple positive activities on mental health and the mediating role of positive emotion. Our research findings can reveal the impact of participation in positive activities on mental health and the mechanism, providing recommendations for promoting mental health.

## 2 Methods

### 2.1 Participants

We used data from the 2021 China General Social Survey (CGSS) which is a national, comprehensive, and large-scale social survey project conducted by the National Research Center of Renmin University of China. The survey was conducted using a multi-stage stratified probability proportionate-to-size (PPS) random sampling method, with counties serving as primary sampling units and urban communities and rural villages as secondary sampling units (28, 29). Post-stratification weights were applied to correct oversampling, ensuring that survey results accurately represented the general population in China (30). A total of 8,148 valid samples were recovered from 320 communities and 19 provinces in the 2021 survey. Representative samples have been recognized and widely used by the academic community.

The inclusion criteria for participants in this study were individuals without missing or abnormal values in positive activity, positive emotion, mental health, and sociodemographic variables. After data sorting and screening, 2581 samples were retained. Among the 2581 participants, the average age was 51.89 ± 17.50, 54.71% were female, 54.9% were agricultural household registration, and 27.20% were unmarried/divorced/widowed. The education level of 33.09% of the participants was primary school and below, 28.28% middle school, 17.98% high school, 7.56% junior college, 11.62% undergraduate, and 1.47% graduate and above. The socioeconomic status of 22.01% of the participants was lower level, 32.97% middle lower level, 38.74 % middle level, 5.70% middle upper level, and 0.58% upper level.

### 2.2 Measures

#### 2.2.1 Positive activities

We measured positive activities by using the question of “The frequency of engaging in the following activities in your free time.” The assignment method is never = 0, several times a year/once a year or less/several times a year or less = 1, several times a month/approximately once a month = 2, 1–2 times a week/several times a week = 3, almost every day/every day = 4, which represents different scores (1, 16, 31). In addition, we classified the types of positive activities in this study into three categories, including physical activities (shopping, participating in physical exercise) (19, 20, 32), social activities (gathering with relatives, meeting with friends) (20, 32), cultural and entertainment activity activities (watching television or videos, going to the cinema, attending cultural events, reading books/newspapers/magazines, listening to music at home, watching live sports, doing crafts, and surfing the internet) (19, 20, 33). The level of participation in various activities was the sum of the scores of included items.

#### 2.2.2 Mental health

According to previous research (16, 34), “In the past 4 weeks, how depressed or frustrated did you feel?” was used to measure mental health. Responses were coded based on a 5-point Likert scale (1 = always, 5 = never). The higher the score, the better the mental health.

#### 2.2.3 Positive emotion

Positive emotion refers to “one's level of pleasurable engagement with the environment” and includes feelings such as “good,” “positive,” “pleasant” (35). In the CGSS database, “Do you feel calm” and “Are you full of energy” are used to measure participants' feelings over the past 4 weeks. Therefore, based on the relevant studies (29) and definition of positive emotion, we used these two questions to measure positive emotion. We coded this variable in a manner consistent with “mental health.” The score of positive emotion is the sum of the scores of two items, with a score range of 2–10.

#### 2.2.4 Control variables

The control variables in this study included age (a continuous variable), gender (female = 0, male = 1), household registration (Agricultural household registration = 0, Non-agricultural household registration = 1), education (Edu, primary school and below = 1, junior high school = 2, high school/technical school/polytechnic school = 3, college = 4, undergraduate = 5, graduate and above = 6), marital status (unmarried/divorced/widowed = 0, cohabitation/first marriage with spouse/remarriage with spouse/separation without divorce = 1), and socioeconomic status (SES, upper level = 5, upper middle level = 4, middle level = 3, lower middle level = 2, lower level = 1).

### 2.3 Statistical analysis

Firstly, we adopted descriptive statistics on positive activities, mental health, positive emotion, and control variables. Secondly, we conducted a correlation analysis between variables. Thirdly, we used Ordinary Least Squares (OLS) to estimate the impact of positive activity on mental health and the three-step method to determine the mediating effect of positive emotion. Before conducting Ordinary Least Squares (OLS) regression, we conducted heteroscedasticity tests and used Robust standard errors regression to address the existing heteroscedasticity issues to obtain more reliable estimation results. The mediating effect was judged based on the criteria proposed by Baron and Kenny (36). According to Baron and Kenny, a mediating effect is determined if the following criteria are met: (a) the predictor variable is significantly correlated with the outcome variable, (b) the predictor variable is significantly correlated with the mediator, and (c) when the predictor variable is included in the model, the mediator variable is significantly correlated with the outcome variable. Finally, we used OLS to analyze the effects of positive activity on mental health in different groups. *p* < 0.05 was considered statistically significant. STATA version 17.0 was used to conduct all statistical analyses.

## 3 Results

### 3.1 Descriptive statistics

The mean of positive activities, physical activities, social activities, and cultural and entertainment activities were 15.83 ± 6.80, 4.43 ± 2.73, 8.40 ± 3.74, and 2.37 ± 1.52, respectively. The mean of mental health and positive emotion were 3.94 ± 1.07 and 7.66 ± 1.71, respectively (see Table 1).

**Table 1:** Descriptive statistics of positive activities, mental health, and positive emotion.

**Variable**	**Category**	**Mean**	**SD**	**Min**	**Max**
Positive activity		15.83379	6.804743	0	42
	Physical activity	3.50988	2.184601	0	8
	Social activity	2.368462	1.515628	0	8
	Cultural and entertainment activity	9.318094	4.508641	0	32
Positive emotion		7.655172	1.709425	2	10
Mental health		3.935684	1.071354	1	5

### 3.2 Correlation analysis

The correlation analysis results show that there is a weak correlation between variables. Positive activity showed a positive correlation with mental health (*r* = 0.1562, *p* < 0.001) and positive emotion (*r* = 0.2409, *p* < 0.001). Physical activities (*r* = 0.1145, *p* < 0.001), social activities (*r* = 0.1199, *p* < 0.001), cultural and entertainment activities (*r* = 0.1108, *p* < 0.001) all showed a positive correlation with mental health. Physical activities (*r* = 0.2043, *p* < 0.001), social activities (*r* = 0.1740, *p* < 0.001), cultural and entertainment activities (*r* = 0.1614, *p* < 0.001) all showed a positive correlation with positive emotion. Positive emotion also showed a positive correlation with mental health (*r* = 0.3993, *p* < 0.001, see Table 2).

**Table 2:** Correlation analysis.

	**Positive activity**	**Physical activity**	**Social activity**	**Cultural and entertainment activity**	**Positive emotion**	**Mental health**
Positive activity	1					
Physical activity	0.6763	1				
	*p* < 0.001					
Social activity	0.5847	0.2710	1			
	*p* < 0.001	*p* < 0.001				
Cultural and entertainment activity	0.8647	0.3612	0.3585	1		
	*p* < 0.001	*p* < 0.0010	*p* < 0.001			
Positive emotion	0.2409	0.2043	0.1740	0.1614	1	
	*p* < 0.001	*p* < 0.001	*p* < 0.001	*p* < 0.001		
Mental health	0.1562	0.1145	0.1199	0.1108	0.3993	1
	*p* < 0.001	*p* < 0.001	*p* < 0.001	*p* < 0.001	*p* < 0.001	*p* < 0.001

### 3.3 The mediating role of positive emotion in the impact of positive activities on mental health

The results of the mediation test are shown in Table 3. In the regression model without mediating variables, it was determined that positive activities positively affected mental health (β = 0.0132, *p* < 0.001). To test the mediating role of positive emotion, it was incorporated into the model. From Table 3, it can be seen that the effect of positive activities on mental health becomes negligible after controlling positive emotion. The positive emotion has a positive effect on the positive activities (β = 0.2281, *p* < 0.001). According to Baron and Kenny, it indicated that positive emotion played a fully mediating role. Similarly, we determined the impact of physical activities, social activities, cultural and entertainment activities on mental health, as well as the mediating role of positive emotion. According to Table 4, physical activities had a positive impact on mental health (β = 0.0321, *p* < 0.01), and positive emotion played a mediating role (β = 0.2288, *p* < 0.001). According to Table 5, social activities had a positive impact on mental health (β = 0.0460, *p* < 0.01), and positive emotion played a mediating role (β = 0.2282, *p* < 0.001). According to Table 6, cultural and entertainment activities have no significant impact on mental health (*p* > 0.05).

**Table 3:** The mediating role of positive emotion between positive activities and mental health.

	**(1)**	**(2)**	**(3)**
	**Mental health**	**Positive emotion**	**Mental health**
Positive activity	0.0132^***^	0.0419^***^	0.0036
	[0.0057, 0.0206]	[0.0291, 0.0547]	[−0.0036, 0.0108]
Positive emotion			0.2281^***^
			[0.2026, 0.2536]
Age	−0.0036^*^	−0.0049^*^	−0.0025
	[−0.0066, −0.0006]	[−0.0096, −0.0003]	[−0.0052, 0.0003]
Gender	0.2184^***^	0.2593^***^	0.1592^***^
	[0.1380, 0.2987]	[0.1320, 0.3867]	[0.0838, 0.2347]
Household registration	0.0751	0.1640^*^	0.0377
	[−0.0177, 0.1680]	[0.0187, 0.3093]	[−0.0489, 0.1243]
Marital status	0.1570^**^	0.0449	0.1467^**^
	[0.0624, 0.2515]	[−0.0990, 0.1887]	[0.0580, 0.2354]
Edu	0.0258	0.0345	0.0179
	[−0.0138, 0.0654]	[−0.0291, 0.0982]	[−0.0185, 0.0543]
SES	0.1505^***^	0.2297^***^	0.0981^***^
	[0.1014, 0.1996]	[0.1546, 0.3048]	[0.0524, 0.1438]
Cons	3.2619^***^	6.4197^***^	1.7975^***^
	[3.0116, 3.5123]	[6.0044, 6.8349]	[1.5069, 2.0881]
*R* square	0.0588	0.0832	0.1803

^*^p < 0.05, ^**^p < 0.01, ^***^p < 0.001.

**Table 4:** The mediating role of positive emotion between physical activities and mental health.

	**(1)**	**(2)**	**(3)**
	**Mental health**	**Positive emotion**	**Mental health**
Physical activity	0.0321^**^	0.1168^***^	0.0054
	[0.0116, 0.0526]	[0.0844, 0.1492]	[−0.0143, 0.0251]
Positive emotion			0.2288^***^
			[0.2032, 0.2544]
Age	−0.0049^***^	−0.0089^***^	−0.0028^*^
	[−0.0078,−0.0020]	[−0.0135,−0.0044]	[−0.0055, 0.0001]
Gender	0.2242^***^	0.2804^***^	0.1601^***^
	[0.1437, 0.3048]	[0.1531, 0.4076]	[0.0843, 0.2359]
Household registration	0.0835	0.1828^*^	0.0417
	[−0.0090, 0.1760]	[0.0392, 0.3265]	[−0.0447, 0.1280]
Marital status	0.1521^**^	0.0278	0.1457^**^
	[0.0575, 0.2466]	[−0.1166, 0.1722]	[0.0569, 0.2344]
Edu	0.0367	0.0650^*^	0.0218
	[−0.0018, 0.0753]	[0.0038, 0.1262]	[−0.0136, 0.0572]
SES	0.1534^***^	0.2342^***^	0.0998^***^
	[0.1047, 0.2021]	[0.1592, 0.3092]	[0.0543, 0.1453]
Cons	3.3877^***^	6.7932^***^	1.8334^***^
	[3.1557, 3.6198]	[6.4213, 7.1651]	[1.5528, 2.1140]
*R* square	0.0581	0.0851	0.1800

^*^p < 0.05, ^**^p < 0.01, ^***^p < 0.001.

**Table 5:** The mediating role of positive emotion between social activities and mental health.

	**(1)**	**(2)**	**(3)**
	**Mental health**	**Positive emotion**	**Mental health**
Social activity	0.0460^**^	0.1169^***^	0.0193
	[0.0169, 0.0751]	[0.0713, 0.1625]	[−0.0078, 0.0464]
Positive emotion			0.2282^***^
			[0.2028, 0.2535]
Age	−0.0040^**^	−0.0068^**^	−0.0024
	[−0.0070, −0.0011]	[−0.0114, −0.0022]	[−0.0052, 0.0003]
Gender	0.2167^***^	0.2554^***^	0.1584^***^
	[0.1363, 0.2971]	[0.1274, 0.3835]	[0.0830, 0.2339]
Household registration	0.0867	0.2096^**^	0.0388
	[−0.0053, 0.1786]	[0.0654, 0.3539]	[−0.0469, 0.1245]
Marital status	0.1630^***^	0.0592	0.1495^***^
	[0.0684, 0.2576]	[−0.0851, 0.2035]	[0.0609, 0.2381]
Edu	0.0393^*^	0.0823^**^	0.0206
	[0.0010, 0.0777]	[0.0216, 0.1429]	[−0.0147, 0.0558]
SES	0.1560^***^	0.2521^***^	0.0985^***^
	[0.1076, 0.2043]	[0.1769, 0.3273]	[0.0533, 0.1436]
Cons	3.3293^***^	6.7107^***^	1.7981^***^
	[3.0876, 3.5710]	[6.3252, 7.0962]	[1.5098, 2.0863]
*R* square	0.0578	0.0742	0.1806

^*^p < 0.05, ^**^p < 0.01, ^***^p < 0.001.

**Table 6:** The mediating role of positive emotion between cultural and entertainment activities and mental health.

	**(1)**	**(2)**	**(3)**
	**Mental health**	**Positive emotion**	**Mental health**
Cultural and entertainment activity	0.0096	0.0288^**^	0.0030
	[−0.0012, 0.0204]	[0.0101, 0.0476]	[−0.0072, 0.0132]
Positive emotion			0.2293^***^
			[0.2040, 0.2547]
Age	−0.0041^**^	−0.0066^**^	−0.0026
	[−0.0071, −0.0010]	[−0.0114, −0.0018]	[−0.0054, 0.0003]
Gender	0.2220^***^	0.2701^***^	0.1600^***^
	[0.1414, 0.3025]	[0.1413, 0.3989]	[0.0845, 0.2356]
Household registration	0.0935^*^	0.2239^**^	0.0422
	[−0.0016, 0.1855]	[0.0788, 0.3690]	[−0.0435, 0.1278]
Marital status	0.1528^**^	0.0321	0.1454^**^
	[0.0580, 0.2476]	[−0.1125, 0.1768]	[0.0566, 0.2342]
Edu	0.0393^*^	0.0790^*^	0.0212
	[0.0005, 0.0781]	[0.0169, 0.1411]	[−0.0146, 0.0570]
SES	0.1596^***^	0.2593^***^	0.1001^***^
	[0.1109, 0.2083]	[0.1839, 0.3348]	[0.0549, 0.1453]
Cons	3.3459^***^	6.7067^***^	1.8079^***^
	[3.0915, 3.6003]	[6.2856, 7.1277]	[1.5123, 2.1034]
*R* square	0.0554	0.0692	0.1800

^*^p < 0.05, ^**^p < 0.01, ^***^p < 0.001.

### 3.4 Grouped regression analysis

According to the results of Table 3, it was found that age, gender, marital status, and SES had a significant impact on mental health. To evaluate the differences between different populations, further grouped regression analysis was conducted. This study divided age into young (18–44), middle-aged (45–59), and older adults (≥60). The regression results showed a positive impact of positive activities on mental health in older adults group (β = 0.024, *p* < 0.001), female (β = 0.015, *p* < 0.01)and male group (β = 0.011, *p* < 0.01), unmarried/divorced/widowed group (β = 0.024, *p* < 0.01), cohabitation/first marriage with spouse/remarriage with spouse/separation without divorce group (β = 0.010, *p* < 0.001), middle (β = 0.013, *p* < 0.05), and upper-middle-level SES group (β = 0.054, *p* < 0.001; Table 7).

**Table 7:** Group regression results.

		**Positive activity**	**Age**	**Gender**	**Household registration**	**Marital status**	**Edu**	**SES**	**Cons**	***R* ^2^**
Age group	Young adults	0.001	-	0.152^*^	−0.104	0.047	0.029	0.113^**^	3.641^***^	0.010
		(0.13)		(2.40)	(−1.52)	(0.66)	(1.06)	(2.79)	(22.54)	
	Middle-aged adults	0.006	-	0.279^***^	0.245^*^	0.267	−0.002	0.198^***^	2.930^***^	0.064
		(0.81)		(3.53)	(2.53)	(1.93)	(−0.05)	(4.18)	(16.08)	
	Older adults	0.024^***^	-	0.213^**^	0.093	0.129	0.071	0.137^***^	2.842^***^	0.076
		(3.97)		(2.94)	(1.11)	(1.51)	(1.73)	(3.33)	(22.01)	
Gender	Female	0.015^**^	−0.003	-	0.050	0.177^**^	0.042	0.137^***^	3.189^***^	0.044
		(2.76)	(−1.30)		(0.73)	(2.64)	(1.41)	(3.91)	(16.25)	
	Male	0.011^**^	−0.004	-	0.102	0.130	0.008	0.169^***^	3.559^***^	0.047
		(2.00)	(-1.91)		(1.58)	(1.80)	(0.28)	(4.69)	(21.01)	
Marital status	unmarried/divorced/widowed=0, cohabit	0.024^**^	−0.001	0.228^***^	−0.017	-	0.018	0.102^**^	3.132^***^	0.059
		(3.00)	(-0.46)	(2.83)	(-0.19)		(0.44)	(2.28)	(11.10)	
	ation/first marriage with spouse/remarriage with spouse/separation without divorce = 1	0.010^***^	−0.004^**^	0.211^***^	0.114^**^	-	0.026	0.170^**^	3.451^***^	0.052
		(2.25)	(−2.27)	(4.31)	(2.01)		(1.09)	(5.60)	(23.74)	
SES	Lower	−0.002	−0.007	0.119	0.184	0.048	0.094	-	3.710^***^	0.031
		(−0.16)	(−1.83)	(1.23)	(1.58)	(0.45)	(1.70)		(11.90)	
	Lower middle	0.011	−0.004	0.224^***^	0.090	0.138	0.020	-	3.643^***^	0.027
		(1.66)	(−1.43)	(3.18)	(1.11)	(1.67)	(0.59)		(16.26)	
	Middle	0.013^*^	−0.002	0.309^***^	0.016	0.259^**^	0.026	-	3.573^***^	0.045
		(2.43)	(−0.98)	(4.98)	(0.22)	(3.23)	(0.87)		(19.00)	
	Upper middle	0.054^***^	0.004	0.118	−0.257	0.102	−0.013		2.962^***^	0.059
		(3.71)	(0.79)	(0.69)	(−1.24)	(0.57)	(−0.18)		(5.76)	
	Upper	−0.064	−0.039^**^	0.968	1.598^**^	−2.317^***^	0.978		6.825^***^	0.349
		(−0.64)	(−3.82)	(1.60)	(3.61)	(−6.76)	(2.29)		(6.90)	

Robust standard errors in parentheses, ^*^p < 0.05, ^**^p < 0.01, ^***^p < 0.001.

## 4 Discussion

### 4.1 Low level of participation in positive activities

In this study, the average of positive activities was 15.83 (with a maximum of 42). A study in the United States reported that the average leisure activity participation level was 24.93 (with a maximum of 50) among adults (23). A study in Japan showed that the average is 17.8 among older adults (with a maximum of 33) (38). Compared to these researches, the average level of positive activity is much lower than the moderate level in our study. The differences in findings may be due to differences in the measurement of positive activities. The measured activities in the study in the United States include physical activities, social activities, spending quiet time alone, spending time unwinding at the end of the day, being in outdoor settings such as gardens, parks, countryside, and so on. In Japanese research, activities measured include physical activity, cultural activity, social activity, as well as travel, creativity, plant cultivation, and so on. The differences in these measurement items may be due to differences in questionnaire design or cultural backgrounds.

### 4.2 Positive activities positively associated with mental health

Our study found that positive activities have a weak protective effect on mental health. Previous studies also found this positive impact. A study including 3,727 participants reported that the more diverse leisure activities older adults participated in, the lower level their depression risk (37). Scholars pointed out that participation in activities creates an environment conducive to mental health which can help individuals increase their self-efficacy and self-confidence, gain companionship and friendship in their interactions, gain social support, and divert attention from unfavorable stimuli, thereby protecting people from psychological problems (17, 19, 37). However, it has also been shown that mental health can in turn affect an individual's participation in positive activities (39). This bi-directional relationship between positive activities and mental health suggests that we need to pay special attention to individuals who may be withdrawn due to psychological problems when encouraging them to engage in positive activities.

Our research found that different types of positive activities have varying impacts on mental health. Physical activities have a positive impact on mental health, which is consist with other studies (19, 40). Similarly, social activities also have a positive impact on mental health, which has been widely confirmed in previous studies (20, 41). Both physical and social activities showed a weak protective effect on mental health. However, in this study, there was no apparent relationship between entertainment and cultural activities and mental health. The research results on the impact of entertainment activities on mental health are inconsistent. Related studies have shown that engaging in excessive entertainment activities such as watching TV, surfing the internet, reading and writing has not been found to be associated with mental health (18, 19). Other studies concluded that participation in more entertainment activities is beneficial to the mental health (20). Some studies shown that the increase in these activities does not affect mental health, but their decrease can become a risk factor for mental health (42). Therefore, although the impact of different types of activities varies, participating in a wider range of activities may be a more beneficial approach for mental health.

### 4.3 Positive emotion played a mediating role

Positive emotion mediated the positive effect of positive activities on mental health. The mediating role of positive emotion has been rarely evaluated in previous empirical studies. A qualitative study reported that participating in various activities can help individuals generate a series of positive emotions and thus improve mental health, which is consistent with our finding (25). There is a view that positive activities can mitigate the negative effects of stressful experiences that threaten physical and mental health, and can buffer stressful experiences by promoting positive emotion related to self-actualization and wellbeing, and therefore they can prevent mental illnesses (43). Previous scholars also have studied the relationship between positive activity and positive emotion, as well as positive emotion and mental health, respectively. A study reported that participating in social interactions can experience stronger positive emotions than being alone, whether the participants take part in face-to-face interactions or technology-mediated communication (44). A study examining the effect of physical activities on life satisfaction validated the mediating role played by positive emotion, and believed that physical activities promoted the body's secretion of dopamine which is closely related to pleasure, thereby increasing an individual's positive emotional experience (26). A research indicated that positive emotion played a mediating role between gratitude and depressive symptoms, which proved that positive emotion can positively affect mental health (45). Our research provided quantitative evidence for the impact of positive activities on mental health through positive emotion, enriching the application of positive activity model, and suggested that positive emotion can mediate the positive effects of physical and social activities on mental health. Our findings suggest that encouraging participation in positive activities and improving positive emotion are important ways to improve mental health.

### 4.4 Further discussion

We divided the samples into several groups for further discussion. Firstly, positive activities had a positive impact on mental health among older adults, which is consistent with relevant studies (46). One possible reason is that older adults are able to gain greater value from leisure activities due to the limited opportunities to participate in society. Secondly, positive activities have a positive impact on the mental health of both male and female group, but in terms of the coefficients, the positive impact on the female group is greater than that on the male population. The relevant research is consistent with our finding (47). This result may be due to the different benefits obtained by males and females in participating in positive activities (20, 47). In groups with different marital status, positive activities have a significant positive impact on both groups, but in terms of the coefficients, the impact is greater in the group without spouse. This may be because individuals without spouses are more likely to pay attention to and enjoy the process of participating in activities, so they can gain more benefits in improving their mental health through participating in activities (48). Finally, we found a positive influence among the middle and upper-middle-level SES population. And our study also indicated that when the socio-economic status is at the top, positive activities didn't have a significant impact on mental health.

This study has some limitations. First, causal inference cannot be made due to the cross-sectional design. Longitudinal studies are needed in the future to explore causal relationships. Second, we are unable to investigate a wider range of positive activities because of the limitation of the database and future research can expand the types of positive activities. Third, in terms of measuring variables, mental health was measured using a single item and the measurement of positive emotion did not use standard scales, which needs to be improved in future research.

## 5 Conclusions

We found that the level of multiple positive activities participation was low, positive activities had a positive impact on mental health, and positive emotion played a mediating role. The impact of positive activities on mental health varies among different types, with physical and social activities having a significant impact on mental health. Our findings also suggested that encouraging participation in positive activities among older adults, female, people without spouse, and middle and upper-middle-income people may lead to better outcomes.

## Data Availability

The original contributions presented in the study are included in the article/supplementary material, further inquiries can be directed to the corresponding author.
